# Comparative Analysis of Whole-Genome Sequence of African Swine Fever Virus Belgium 2018/1

**DOI:** 10.3201/eid2506.190286

**Published:** 2019-06

**Authors:** Jan H. Forth, Marylène Tignon, Ann Brigitte Cay, Leonie F. Forth, Dirk Höper, Sandra Blome, Martin Beer

**Affiliations:** Friedrich-Loeffler-Institut, Greifswald, Germany (J.H. Forth, L.F. Forth, D. Höper, S. Blome, M. Beer);; Sciensano, Brussels, Belgium (M. Tignon, A.B. Cay)

**Keywords:** African swine fever virus, whole-genome sequencing, molecular epidemiology, viruses

## Abstract

We analyzed the whole-genome sequence of African swine fever virus Belgium 2018/1. The strain fits into the European genotype II (>99.98% identity). The high-coverage sequence revealed 15 differences compared with an improved African swine fever virus Georgia 2007/1 sequence. However, in the absence of genetic markers, no spatial or temporal correlations could be defined.

African swine fever (ASF) is one of the most pathogenic viral diseases of swine leading to clinical and pathomorphological signs of a viral hemorrhagic fever (*1*). In 2007, ASF was introduced into Georgia (*2*) and thereafter into numerous eastern European and European Union (EU) countries (*3*), as well as Asia (China, Mongolia, and Vietnam) (World Organization for Animal Health–World Animal Health Information database, http://www.oie.int/wahis_2/public/wahid.php/Wahidhome/Home/indexcontent, 2019 Feb 21).

In September 2018, the African swine fever virus (ASFV) was introduced into Belgium (*3*,*4*); as of April 28, 2019, 719 cases have been reported by Belgium’s Federal Agency for the Safety of the Food Chain (http://www.afsca.be/ppa/actualite/belgique).

Samples from the first 2 cases were taken for analysis to the Belgium National Reference Laboratory for ASF at Sciensano, Brussels, and were later confirmed by the ASF EU Reference Laboratory (EURL) in Valdeolmos, Spain. Partial sequencing at the EURL revealed a p72 genotype II with CVR-1, IGR-2, and MGF1 variants. Initial assessments of virus type and epidemiology were published by Garigliany et al. (*4*) to share the data without delay. Subsequently, samples were transferred to the Friedrich-Loeffler-Institut, Greifswald, Germany, for whole-genome sequencing.

We prepared samples and sequenced them on an Illumina MiSeq (https://www.illumina.com), as previously described (*5*). In addition, we enriched DNA libraries for Illumina sequencing for ASFV specific target sequences using an ASFV-specific myBaits kit (Arbor Biosciences, https://arborbiosci.com). We analyzed the resulting sequence data by mapping against an improved ASFV Georgia 2007/1 sequence (International Nucleotide Sequence Database Collaboration accession no. FR682468.2) using Newbler 3.0 software (*6*); we assembled the identified ASFV-specific reads using SPAdes 3.13.0 (*7*).

For the assembly of the inverted terminal repeat (ITR) regions, we mapped the reads against the individual ASFV-Georgia 2007/1 ITR regions using Newbler and manually assembled them with the ASFV Belgium 2018/1 sequence in Geneious 11.1.5 (Biomatters, https://www.geneious.com). For validation, we mapped all reads along the final contig using Newbler; the result was a median unique depth of 292 with an interquartile range of 42. We annotated the sequence according to the improved ASFV Georgia 2007/1 sequence, using Glimmer3 in Geneious. We aligned different available ASFV whole-genome sequences using MAFFT 7.388, and we performed a phylogenetic analysis using IQ-TREE v1.6.5 (*8**,**9*). The whole-genome sequence is available from the International Nucleotide Sequence Database Collaboration databases under study accession no. PRJEB31287 and sequence accession no. LR536725.

The ASFV Belgium 2018/1 whole-genome sequence has a length of 190,599 bp. Comparison with the improved ASFV Georgia 2007/1 sequence revealed 15 differences, for an overall sequence identity of 99.98% at the nucleotide level. The detected differences included 4 nucleotide transitions, 5 nucleotide transversions, 5 changes in homopolymer regions, and 1 integration of a repeat into a previously described variable intergenic region (*10*) (Table).

**Table:** Differences in the whole-genome sequence of ASFV-Belgium2018/1 and the improved ASFV-Georgia2007/1 reference genome (indicated in bold and underlined)*

Position	Type	Difference	ASFV Georgia 2007/1†	ASFV Belgium 2018/1†	Coverage	Gene	Synonymous/ nonsynonymous	ORF change
121	Transversion	G121C	AAGAT	AA**C**AT	44	ITR	NA	NA
129	Transversion	A129T	AAATA	AA**T**TA	47	ITR	NA	NA
479	Variation	+T	9 × T	10 × T	767	ITR	NA	NA
1393	Variation	+C	9 × C	10 × C	1375	NA	NA	NA
6786	Variation	–T	9 × T	8 × T	1755	NA	NA	NA
7061	Transition	C7061T	CCCAG	CC**T**AG	1556	MGF 110–1L	Trp197Stop	Shortened by 18 codons
15688	Variation	+3C	17 × C	20 × C	127	MGF 110–13Lb	Frameshift	Enlarged by 1 codon
17640	Variation	+2G	10 × G	12 × G	1117	NA	NA	NA
17854	Variation	+G	9 × G	10 × G	1304	NA	NA	NA
19807	Variation	–G	7 × G	6 × G	1238	NA	NA	NA
20017	Variation	–2G	16 x G	14 × G	832	ASFV G ACD 00350	Frameshift	Truncation
21815	Variation	+2G	9 × G	11 × G	1184	NA	NA	NA
27439	Variation	–T	10 × T	9 × T	1895	NA	NA	NA
44586	Transition	A44586G	TGAAA	TG**G**AA	1292	MGF 505–9R	Lys323Glu	NA
73282	Variation	–T	9 × T	8 × T	1241	NA	NA	NA
134524	Transition	T134524C	CATTT	CA**C**TT	1665	NP419L	Asp414Ser	NA
145080	Transition	G145080A	GAGGC	GA**A**GC	1266	D117L	Pro84Leu	NA
170827	Transversion	T170827A	GATGG	GA**A**GG	1263	I267L	Ile195Phe	NA
190138	Variation	+A	9 × A	10 × A	3021	DP60R	Frameshift	22 changed codons
173402	Repeat integration	+TATATAGGAA	NA	+TATATAGGAA	1731	NA	NA	NA
190478	Transversion	T190478A	TATTT	TA**A**TT	49	ITR	NA	NA
190486	Transversion	C190486G	ATCTT	AT**G**TT	49	ITR	NA	NA

*Differences are show in bold and underlined. ASFV, African swine fever virus; ITR, inverted terminal repeat; NA, not applicable; ORF, open reading frame. †International Nucleotide Sequence Database Collaboration accession nos: ASFV Georgia 2007/1, FR682468.2; ASFV Belgium 2018/1, LR536725.

Altogether, 4 differences in annotated genes are nonsynonymous; 2 cause a frameshift, thereby truncating the MGF 110–1L gene and changing the amino acid sequence of the DP60R protein; and 9 differences were identified in noncoding regions (Table). The differences in the ITR regions must be viewed carefully because of the low coverage in these particular parts of the genome, but the differences in the core regions are well supported by the sequencing data (Table). The differences at the specific positions 7,061; 44,586; 134,524; and 170,827 were also identified in the ASFV-SY2018 (China), Estonia 2014, Kashino 04/13 (Russia), and Pol 16/17 (Poland) sequences, and position 170,827 also in ASFV strain Odintsovo_02/14 (Russia). Further genetic differences could be identified in 7 so-called poly G/C regions. Whether these are artifacts originating from sequencing any of the analyzed genomes or pose real differences remains to be determined by the analysis of further sequences from Belgium and other countries, which is in progress.

Finally, the alignment of all publicly available eastern European whole-genome sequences, as well as ASFV-China, shows that all these genomes are nearly identical with identities of more than 99.9% (Appendix Figure).

In conclusion, we provide a whole-genome analysis of ASFV from Belgium, which could show a high overall identity to recent ASFV strains from eastern Europe and China. We also identified locations showing differences from ASFV Georgia 2007/1 in single nucleotides, as well as a previously described repeat insertion. However, because the low mutation rate and the corresponding high genetic stability of the eastern European ASFV strains have hindered the definition of reliable genetic markers thus far, the currently available whole-genome information does not allow for further statements regarding correlations in space and time, and does not provide enough evidence for a more detailed mapping of strain origin.

Although MGF110 was assigned a possible function in preparing the endoplasmic reticulum for viral morphogenesis (*11*), in the absence of any observations regarding virus attenuation in the field, no conclusion can be drawn on the effect of the observed differences. Therefore, further in vitro and in vivo characterizations using the ASFV Belgium 2018/1 isolate are needed.

In the future, more high-quality whole-genome ASFV sequences might allow identification of genetic markers that could aid high-resolution molecular epidemiology. Coordinated efforts to improve data sharing, together with harmonized protocols under quality assurance, are of utmost importance to interpret results correctly and aid the fight against ASF.

Appendix Phylogenetic tree of all available African swine fever virus whole-genome sequences.
