# Supplementary material for: Comparative Analysis of Whole-Genome Sequence of African Swine Fever Virus Belgium 2018/1
Source: Emerg Infect Dis. 2019 Jun;25(6):1249–52. doi: 10.3201/eid2506.190286 (PMC6537744; doi:10.3201/eid2506.190286)
Supplement: Appendix — Phylogenetic tree of all available African swine fever virus whole-genome sequences. [file 19-0286-Techapp-s1.pdf]

Article DOI: https://doi.org/10.3201/eid2506.190286

Comparative Analysis of Whole-Genome Sequence of African Swine Fever Virus Belgium 2018/1

Appendix

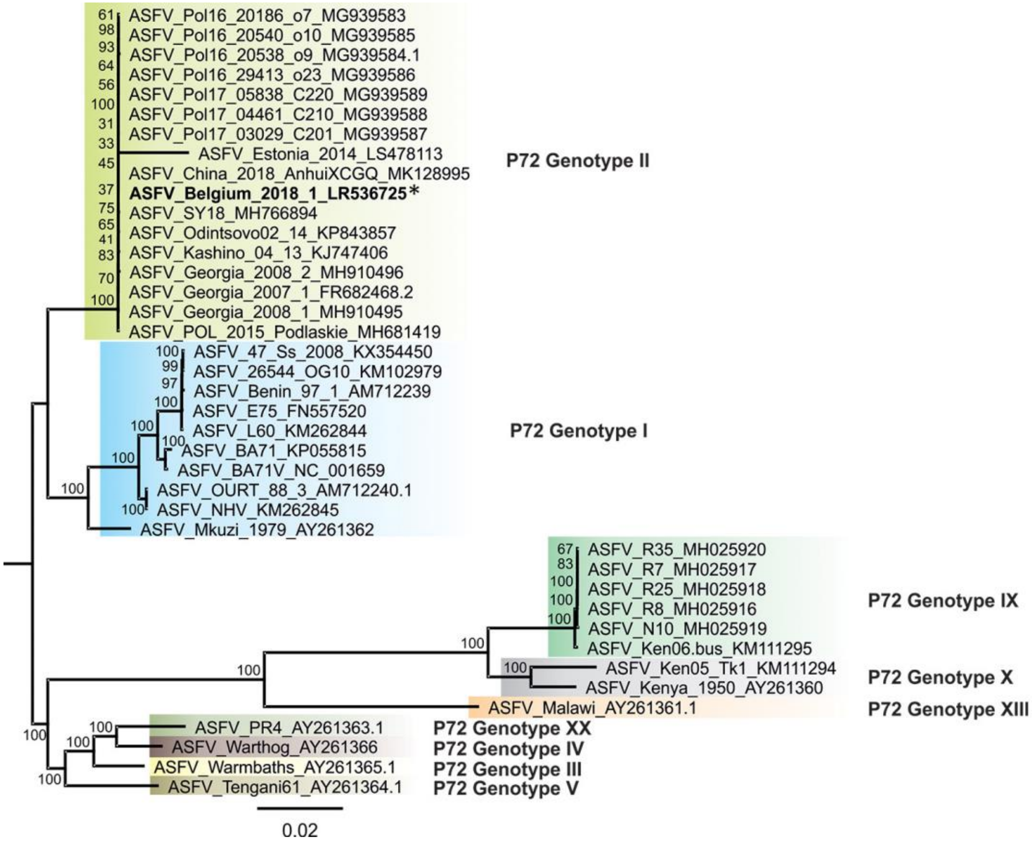

Appendix Figure. Phylogenetic tree of all available African swine fever virus (ASFV) whole-genome sequences. We constructed a maximum-likelihood tree using IQ-TREE v1.6.5 (www.iqtree.org) with standard model selection, resulting in the best-fit model K3Pu+F+R3 (3 substitution types model + empirical base frequencies + FreeRate model with 3 categories) based on MAFFT v7.388 (https://mafft.cbrc.jp) aligned ASFV whole-genome sequences. Statistical support of 10,000 ultrafast bootstraps is indicated at the nodes. Taxon names include, where available, ASFV designation and International Nucleotide Sequence Database Collaboration accession number. Numbers along branches are bootstrap values. Scale bar represents nucleotide substitutions per site. Colors indicate specific genotypes; * indicates the genome discussed in this study.
